# Hormonal Dysregulation and Neuroinflammation in Endometriosis: Convergent Druggable Pathways

**DOI:** 10.3390/cimb48050528

**Published:** 2026-05-19

**Authors:** Ioana-Laura Olteanu, Ciprian Pușcașu, Corina Andrei, Anca Zanfirescu

**Affiliations:** Faculty of Pharmacy, “Carol Davila” University of Medicine and Pharmacy, Traian Vuia 6, 020956 Bucharest, Romania; ioana-laura.olteanu@drd.umfcd.ro (I.-L.O.); anca.zanfirescu@umfcd.ro (A.Z.)

**Keywords:** endometriosis, hormonal dysregulation, neuroinflammation, estrogen dominance, progesterone resistance, pain mechanisms, nociceptor sensitization, central sensitization, neuroimmune interactions

## Abstract

Endometriosis is a chronic, estrogen-dependent disorder defined by ectopic endometrial-like tissue growth, persistent inflammation, and aberrant innervation. Emerging evidence indicates that disease progression and symptom severity are driven by a reciprocal interaction between hormonal dysregulation and neuroinflammatory signaling. This narrative review synthesizes human-based mechanistic and clinical evidence on the hormonal–neuroinflammatory interface in endometriosis, drawing on peer-reviewed publications retrieved from PubMed and Scopus through November 2025. The publications comprised studies using data from patient-derived tissues, primary endometriotic cells, and clinical cohorts. Several convergent molecular nodes at this interface were identified: the prostaglandin E_2_–prostaglandin E receptor 2/prostaglandin E receptor 4–aromatase axis, estrogen receptor beta—nuclear factor kappa B signaling, interleukin-6/signal transducer and activator of transcription 3-mediated fibrosis, neurotrophin pathways, transient receptor potential channels (TRPV1/TRPA1), and neurokinin 1 receptor signaling. In this integrated model, endocrine dysfunction fuels neuroinflammation, which in turn impairs steroid responsiveness. This cycle explains the frequent pain–lesion mismatch and the persistence of symptoms despite standard hormonal suppression. Targeting these druggable interface pathways enables better patient stratification and more effective combination therapies for endometriosis.

## 1. Introduction

Endometriosis is increasingly recognized as a complex, chronic inflammatory disease whose pathophysiology extends beyond the ectopic localization of endometrial-like tissue, encompassing systemic immune dysregulation, neurogenic inflammation, and central pain sensitization [1,2,3]. Despite being a leading cause of chronic pelvic pain and infertility, it remains an enigma of modern gynecology [1]. While traditional research has centered on estrogen dominance, the high rate of non-response to hormonal therapy demands a more integrated perspective. Endometriosis is increasingly recognized as a disease driven by the synergy of systemic hormonal dysregulation, epigenetic reprogramming, and a self-perpetuating neuroinflammatory microenvironment [2,4].

The core of the problem lies in the transition from an estrogen-dependent state to an autonomous, inflammatory one. In ectopic lesions, classical hormonal signaling is disrupted: although frontline progestins are effective in approximately two-thirds of patients, the remaining one-third exhibit progesterone resistance, and autonomous ‘intracrine’ estrogen production further bypasses hormonal suppression, fueling persistent lesion survival [5,6]. This hormonal chaos directly feeds a specialized neuroinflammatory cascade. In this environment, immune cells such as macrophages and mast cells actively “rebuild” the local nervous system through ectopic neurogenesis [7,8,9]. This bidirectional crosstalk between endocrine dysregulation and peripheral nerve sensitization facilitates the transition from focal ectopic implantation to a systemic chronic pain state.

This review explores these convergent pathways, moving beyond the superficial “estrogen-only” model. By dissecting the molecular bridges between endocrine failure and neural hypersensitivity, we aim to identify specific “druggable” targets, ranging from epigenetic modulators to kinase inhibitors, which could ultimately provide a more personalized and effective therapeutic roadmap for patients who have exhausted conventional options.

## 2. Physiological Steroid Hormone Signaling in the Endometrium

The human endometrium is a highly steroid-responsive tissue in which epithelial, stromal, immune, and vascular compartments are dynamically remodeled across the menstrual cycle in response to ovarian estradiol (E2) and progesterone (P4) [10,11,12]. Steroid hormones regulate endometrial function primarily through receptor-mediated genomic mechanisms [13,14]. Estrogen signaling in the endometrium is mediated primarily through estrogen receptors ERα and ERβ [15]. ERα-regulated signaling predominates during the proliferative phase and extends into the early secretory phase [16,17]. In the absence of estrogen, ERα is maintained in a transcriptionally competent state through association with chaperone complexes, including heat-shock proteins [18,19,20,21,22]. Estrogen binding induces receptor dissociation from chaperones, dimerization, and nuclear translocation, initiating genomic signaling via direct binding to estrogen-response elements or through tethering to other transcription factors such as activator protein-1, specificity protein-1, and nuclear factor kappa-B (NF-κB) [15,23].

During the proliferative phase, ERα signaling drives epithelial expansion through stromal paracrine mediators. This process is largely mediated by the IGF-1/PI3K/AKT/mTOR pathway, with additional contributions from fibroblast growth factors and CCAAT/enhancer-binding protein beta [24,25,26,27,28]. During the secretory phase, ERα supports decidual priming and embryo implantation by modulating endometrial receptivity programs. Key downstream effectors include mucin 1 and the leukemia inhibitory factor/Janus kinase/signal transducer and activator of transcription 3 (STAT3)/early growth response 1 transcriptional axis [29,30,31,32].

P4 signaling becomes dominant during the secretory phase and decidualization, underscoring the functional interplay between E2 and P4 pathways [16,24]. P4 acts primarily through the P4 receptor (PR) isoforms, PR-A and PR-B [33]. PR-A is considered essential for uterine function and fertility, whereas PR-B is required for mammary gland development and can promote uterine epithelial proliferation when PR-A-mediated repression is absent [34]. PR plays a central role in steroid-dependent regulation by forming a feedback loop with E2 signaling: E2 induces PR expression via ERα, while PR-B suppresses ERα signaling primarily by inhibiting its transcriptional activity, with additional context-dependent effects on its expression [34].

Canonical PR signaling also involves ligand-induced receptor activation, dimerization, and nuclear translocation, followed by binding to P4 response elements and recruitment of transcriptional co-regulators, particularly members of the p160/SRC family [35,36]. PR also regulates transcription indirectly through interactions with DNA-bound transcription factors and STAT3 [37].

A well-defined epithelial–stromal paracrine cascade downstream of PR signaling is initiated by epithelial Indian hedgehog, which induces stromal chicken ovalbumin upstream promoter–transcription factor II [38]. COUP-TFII suppresses E2-driven epithelial proliferation and promotes stromal bone morphogenetic protein 2, which subsequently induces Wnt family member 4 (WNT4) to support Wnt/β-catenin-dependent implantation and decidualization [39,40].

In parallel, PR regulates additional stromal mediators that contribute to uterine receptivity, including homeobox protein A10 and heart and neural crest derivatives expressed 2, which are PR-dependent but not part of the above-mentioned signaling cascade [41,42,43,44].

Although E2 and P4 are the primary regulators of endometrial function, emerging evidence indicates that androgen signaling also contributes to the fine-tuning of endometrial physiology. Androgen signaling is mediated predominantly through the androgen receptor (AR), a ligand-activated nuclear transcription factor [45]. AR expression is highly cycle-dependent in the human endometrium, with the highest levels during the proliferative phase, predominantly in stromal nuclei, followed by a decline in the secretory phase [46,47]. AR expression is upregulated by androgens and estrogens and suppressed by progestins, and it increases with PR antagonism [12,45]. Human in vitro data suggests that androgens inhibit endometrial proliferation, supraphysiological testosterone being linked to endometrial atrophy [48]. During the secretory phase, locally produced androgens may support AR-dependent immune and vascular remodeling to regulate decidualization and receptivity [49].

In addition to classical genomic mechanisms, all major steroid hormone receptors can elicit rapid non-genomic signaling responses. A subset of ERα localized at the plasma membrane activates signaling pathways such as phospholipase C beta (PLCβ)-dependent Ca^2+^ mobilization, extracellular signal-regulated kinase (ERK)/mitogen-activated protein kinase (MAPK), PI3K, and adenylate cyclase–cyclic adenosine monophosphate (cAMP) signaling, thereby modulating uterine sensitivity to E2 [50,51,52,53].

Beyond classical estrogen receptors, the G protein–coupled estrogen receptor contributes to estrogen signaling, although its role in endometrial physiology appears limited [24,54]. Similarly, P4 can induce rapid non-genomic responses via membrane-associated receptor systems distinct from classical PRs, including progesterone receptor membrane component and adipoQ receptor family members [24].

## 3. Hormonal Dysregulation in Endometriosis

### 3.1. Dysregulated Estrogen Signaling and Estrogen Dominance

Excessive E2 signaling is a central driver of lesion growth and persistence in endometriosis, driven by increased local E2 production and elevated ERβ expression.

Intralesional E2 level increases due to a local elevated expression of the E2-synthesizing enzyme cytochrome P450 aromatase (CYP19A1), together with reduced expression of 17β-hydroxysteroid dehydrogenase type 2 (17β-HSD2) [55,56,57,58]. Under physiological conditions, 17βHSD2 is induced by P4 and catalyzes the conversion of E2 to the less potent estrone. However, its expression is diminished in P4-resistant endometrial tissue [59].

ERβ is overexpressed in endometriotic stromal cells [60]; this is accompanied by reciprocal downregulation of ERα, resulting in a markedly increased ERβ-to-ERα ratio. Elevated nuclear ERβ in glandular cells across lesion types indicates a central role for ERβ-driven transcription in the endometriotic epithelium [55,61]. Higher ERβ levels in ovarian and fallopian tube lesions compared with peritoneal disease indicate site-specific estrogen responsiveness and heterogeneous inflammatory activity across lesion subtypes [60,62]. In contrast, stromal ERβ shows little variation across sites, consistent with more conserved regulatory mechanisms.

Consistent with these findings, an increased *ESR2*-to-*ESR1* ratio, particularly in ovarian lesions, reflects the shift toward ERβ-dominant signaling associated with a pro-proliferative and pro-inflammatory transcriptional profile. This ratio is also physiologically higher in the secretory than in the proliferative phase in both patients and controls, suggesting normal cycle-dependent modulation of ERβ that may be exaggerated in endometriosis [62].

### 3.2. Progesterone

While excessive E2 signaling is a well-established driver of lesion growth and persistence, alterations in P4 signaling are equally important in endometriosis pathophysiology. Changes in P4 receptor expression and isoform balance contribute to progesterone resistance in up to one-third of symptomatic women, despite normal circulating hormone levels. This resistance is characterized by impaired P4 receptor activation and reduced transcription of P4-responsive genes. Because P4 normally counteracts E2-driven proliferation and supports decidualization, reduced P4 responsiveness promotes lesion progression and impairs endometrial receptivity [63].

Early studies reported loss of PR-B with preserved PR-A expression in peritoneal lesions, consistent with a PR-A-dominant, transcriptionally constrained state [64]. Subsequent analyses revealed lesion-specific heterogeneity: peritoneal lesions exhibit predominant PR-A expression, whereas ovarian lesions and eutopic endometrium retain both isoforms but remain PR-A-skewed [65]. This imbalance is frequently reinforced by PR-B promoter hypermethylation in microdissected epithelial compartments of endometriotic implants, indicating stable epigenetic repression despite ligand availability [66].

Accordingly, PR-B is generally reduced across endometriotic lesions, resulting in PR-A predominance, although the extent of PR-B loss varies by lesion type [5]. In deep infiltrating endometriosis (DIE), overall PR expression is further diminished and may decline in response to P4 treatment [67], while the levels of HSD17B1 increase. Together, these findings support a model of progesterone resistance or altered progesterone responsiveness, particularly in stromal DIE cells, indicating variability across lesion types and cellular compartments.

Reduced PR expression in endometriotic tissue results from the interplay of several mechanisms. Estrogen regulates PR expression in the endometrium. Under physiological conditions, E2 induces PR expression via ERα, priming the endometrium for P4 responsiveness during the secretory phase. In endometriosis, however, estrogen imbalance disrupts this regulation [68,69]. Suppression of ERα activity by ERβ reduces PR expression, particularly the PR-B isoform, despite sustained or elevated local estrogen levels. Estrogen excess further contributes to P4 resistance through epigenetic and transcriptional mechanisms [66,70], lowering total PR levels and shifting the PR-A/PR-B balance toward PR-A predominance. This reinforces a feed-forward loop in which impaired P4 responsiveness allows continued estrogen-driven proliferation, inflammation, and lesion persistence [71].

Epigenetic mechanisms play a central role in regulating PR expression and responsiveness in endometriosis. DNA methyltransferases (DNMTs) have been implicated, although reported expression patterns are inconsistent. Some studies show increased DNMT1, DNMT3A, and DNMT3B in ectopic epithelial cells, with DNMT3A elevated in eutopic endometrium and DNMT1 enriched in lesions [72]. In stromal cells, persistent DNMT3B enrichment at the *ESR1* promoter suggests a role in *ESR1* hypermethylation and repression [72,73,74]. In contrast, other studies report reduced DNMT expression in both ectopic and eutopic tissues, particularly in the secretory phase [75,76].

Recent evidence demonstrates that DNA methylation profiles capture a significant proportion of endometriosis risk beyond common genetic variants, highlighting an independent and complementary contribution of epigenetic variation to disease [77]. In addition, distinct combinations of genetic and epigenetic alterations may underlie variable disease phenotypes, with DNA methylation changes occurring partly independent of underlying genetic variation and contributing to lesion heterogeneity [78]. These discrepancies likely also reflect the context-dependent nature of DNA methylation in endometriosis, which varies according to tissue type, menstrual cycle phase, and cellular composition, supporting a polyepigenetic model rather than a single uniform methylation signature [79]. Despite this variability, functional data support a pathogenic role for DNMT3A and DNMT3B, linking them to activation of proliferative pathways and estrogen-dependent lesion growth [80,81].

Non-coding RNAs (ncRNAs) add another layer of regulation by modulating gene expression at transcriptional and post-transcriptional levels. Competitive endogenous RNA networks involving long non-coding RNAs (lncRNAs), microRNAs (miRNAs), and messenger RNAs (mRNAs) fine-tune gene programs relevant to implantation and endometrial receptivity and are disrupted in endometriosis [82,83,84,85,86].

miRNAs contribute directly to progesterone resistance by repressing PR expression or its downstream signaling. For example, miR-196a and miR-194-3p reduce PGR expression and impair decidualization, while others (including miR-92a, miR-297, miR-143-3p, and miR-21-5p) act through pathways such as PTEN, YAP1, and transforming growth factor beta 1 (TGF-β1) signaling [87,88,89]. Additional miRNAs target progesterone-responsive genes, such as homeobox protein A10, further compromising receptivity and decidualization [90,91]. Some also promote lesion invasiveness and epithelial–mesenchymal transition [92,93]. Importantly, circulating miRNA signatures with high diagnostic accuracy have been identified in women with endometriosis, supporting their utility as non-invasive biomarkers [94,95].

LncRNAs similarly regulate steroid-responsive pathways and disease progression. lncRNA H19 is linked to infertility, disease severity, and impaired receptivity through interactions with let-7 microRNAs and regulation of IGF-1 receptor and integrin β3 [96,97]. Other lncRNAs modulate hormone signaling, chromatin organization, and cellular processes such as proliferation and invasion [98]. In addition, widespread N6-methyladenosine modifications of lncRNAs have been observed in ovarian endometriosis, implicating RNA methylation in disease progression [99].

In endometriosis, P4 resistance primarily reflects post-receptor defects rather than insufficient hormone availability. In addition to PR-A predominance and relative PR-B deficiency, impaired signaling arises from altered co-regulator availability, repressive chromatin states, and hyperactivation of MAPK/ERK and PI3K/AKT pathways, which destabilize PR function and downstream transcription [88,89]. Several recurrent post-receptor defects have been identified. Stromal expression of key PR co-regulators, including Hic-5, Kruppel-like factor 9, and FK506-binding protein 52, is reduced, impairing progesterone responsiveness and disrupting PR-dependent signaling pathways such as WNT/dickkopf WNT signaling pathway inhibitor 1 (DKK1) [100,101,102,103].

Kinase-driven post-translational mechanisms further limit receptor and effector function. Elevated ERK1/2/AKT activity promotes phosphorylation-dependent degradation of progesterone receptor and forkhead box O1 (FOXO1), reduces nuclear localization, and impairs decidual gene expression. Although inhibition of these pathways can partially restore cell-cycle control, differentiation programs remain incompletely rescued, indicating selective uncoupling of decidualization [104,105,106]. Inflammatory signaling via STAT3 and NF-κB reinforces this resistant state [107].

These molecular defects converge on a common phenotype of impaired decidualization, characterized by reduced insulin-like growth factor binding protein 1 and prolactin expression, loss of nuclear FOXO1, persistent cell proliferation, and sustained inflammation. Disruption of nuclear trafficking further contributes: FKBP52 deficiency enhances lesion growth and inflammation in experimental models and is reduced in human disease, including during the window of receptivity in primates [102,108].

Finally, altered chromatin accessibility and transcription factor cooperation limit PR function. Reduced CXXC finger protein 1 is associated with decreased expression of PR-responsive genes such as *GATA2*, *SOX17*, and *IHH* [109]. GATA2 itself is diminished and uncoupled from progesterone receptor expression, while loss of AT-rich interaction domain 1A, a chromatin remodeling component that interacts with PR, increases lesion burden in experimental models [110,111]. Together, these findings underscore that receptor presence alone is insufficient without permissive chromatin and appropriate partner-factor recruitment.

Overall, P4 resistance in endometriosis arises from multilayered post-receptor dysfunction involving receptor isoform imbalance, defective co-regulator assembly, altered nuclear trafficking, epigenetic repression, and kinase-driven signaling. These insights support therapeutic strategies that combine progestins with agents targeting kinase activity or epigenetic regulation, tailored to lesion type and disease severity [112].

### 3.3. Androgen

In endometriosis, AR signaling becomes heterogeneous and strongly context-dependent [113]. AR is predominantly stromal, detectable across lesion types, with higher stromal density in pelvic lesions than in ovarian endometriomas, and preserved expression in DIE, even after progestin treatment [113,114]. Within lesions, androgen metabolism is compartment-specific: perivascular cells show reduced AR and 5α-reductase, while stromal cells overexpress steroid-metabolizing enzymes [115]. In eutopic endometrium, AR mRNA levels are often unchanged, but AR target genes are dysregulated, indicating disruption at the signaling level rather than receptor abundance [80].

A key feature is the disconnect between circulating and local androgens. Intralesional testosterone levels are markedly elevated relative to serum levels, alongside altered expression of steroidogenic enzymes, including increased HSD3B2 and reduced CYP11A1 [116]. Although serum testosterone is often lower in affected women and may be linked to disease risk [117,118], both AR and 5α-reductase are expressed in lesions, supporting local dihydrotestosterone production [114]. Thus, circulating levels do not reflect tissue-level androgen activity.

AR function is further shaped by epigenetic and post-transcriptional regulation. AR repression has been reported in reproductive tissues [119], while PI3K/AKT signaling influences AR localization and activity [117]. Changes in co-regulators, such as steroid receptor coactivator 1, may further shift AR, ER, and PR signaling balance [120,121]. Although androgens influence immune and inflammatory pathways, lesion-level evidence linking AR activity to defined immune phenotypes remains limited [122]. AR also does not appear to be a reliable marker of progestin response, as progestins suppress ERα without altering AR expression in deep endometriosis [123].

Collectively, the evidence supports a spatial and functional “uncoupling” model of AR biology in endometriosis: androgen availability, AR expression, and AR transcriptional output frequently diverge across lesions and cellular compartments. This divergence—shaped by intracrine steroid metabolism, epigenetic repression, signaling crosstalk, and co-regulator context—likely contributes to inflammation, lesion persistence, infertility, and variable therapeutic response.

**Figure 1:**
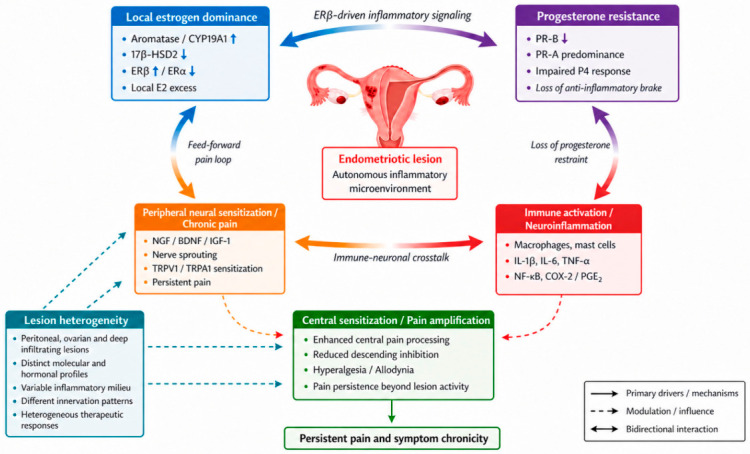
Hormonal–neuroinflammatory feed-forward loop in endometriosis. Legend: ERα, Estrogen receptor alpha; ERβ, Estrogen receptor beta; E2, Estradiol; 17β-HSD2, 17-beta-hydroxysteroid dehydrogenase type 2; PR-A, Progesterone receptor A; PR-B, Progesterone receptor B; P4, Progesterone; IL-1β, Interleukin-1 beta; IL-6, Interleukin-6; TNF-α, Tumor necrosis factor alpha; NF-κB, Nuclear factor kappa B; COX-2, Cyclooxygenase-2; PGE2, Prostaglandin E2; NGF, Nerve growth factor; BDNF, Brain-derived neurotrophic factor; IGF-1, Insulin-like growth factor 1; TRPV1, Transient receptor potential vanilloid 1; TRPA1, Transient receptor potential ankyrin 1; TrkA, Tropomyosin receptor kinase A; STAT3, Signal transducer and activator of transcription 3.

These findings support a model in which neuroinflammatory and pain pathways are tightly coupled to local hormonal dysregulation, as summarized in Figure 1.

## 4. Neuroinflammation and Pain in Endometriosis: Mechanisms and Phenotypic Variation

### 4.1. Ectopic Neurogenesis and Lesion Innervation

Endometriotic lesions are abnormally innervated by sensory nerve fibers, a phenomenon termed ectopic neurogenesis. Lesions contain an increased density of small-caliber fibers, predominantly unmyelinated C-type sensory neurons, compared with normal tissues, including peritoneum and eutopic endometrium from women without endometriosis [124,125,126]. Nerve fiber density correlates with pain severity: women with pelvic pain exhibit hyperinnervated lesions, with sensory fibers located closer to ectopic endometrial glands than in lesions from women without pain [127,128]. These ectopic fibers transmit aberrant nociceptive signals to spinal nerve roots, amplifying central pain processing. This could provide a mechanistic explanation for the disproportionate pain burden associated with DIE, which has a limited size, but displays substantially higher innervation density than superficial peritoneal lesions [129].

Nerve growth factor (NGF) and brain-derived neurotrophic factor (BDNF) show elevated serum levels and are overexpressed in endometriotic lesions, including ovarian and deeply infiltrating lesions, compared with normal endometrium, contributing to lesion-associated neuroproliferation and pain [130,131,132]. Thus, BDNF/TrkB expression in both lesions and eutopic endometrium is significantly associated with dysmenorrhea pain scores, with BDNF being proposed as a biomarker of central sensitization in a subset of patients. Similarly, elevated NGF levels are detected in peritoneal lesions from women with severe pain, including deep dyspareunia [133]. Experimental blockade of the NGF–TrkA pathway reduces endometriosis-associated pain [134]. Collectively, NGF and BDNF promote local hyperinnervation, sensitization of ectopic sensory endings, and amplification of nociceptive signaling to the central nervous system.

### 4.2. The Immune–Neuronal Interface

Endometriotic lesions are characterized by chronic inflammation and dense immune–neuronal interactions. Macrophages and mast cells accumulate in lesions and in peritoneal fluid, where they interact closely with sensory nerve fibers [7,135,136]. Despite impaired phagocytic function, macrophages secrete cytokines [137,138,139,140,141], neurotrophic factors [136,142], and IGF-1, collectively promoting nerve growth and sensitization [8].

Mast cells further link inflammation to pain through increased infiltration and degranulation. They release mediators such as histamine, cytokines (TNFα, IL-1β, IL-6, IL-8), proteases, and prostaglandins, which directly activate nociceptors and drive inflammatory hyperalgesia [143,144,145,146,147]. In turn, neuropeptides from sensory fibers (substance P, calcitonin gene-related peptide) activate mast cells, creating a feed-forward cycle of neuroinflammation and pain. Mast cell density correlates with pain severity [122].

Pro-inflammatory cytokines, such as IL-1β, IL-6, and TNF-α, are increased in lesions and peritoneal fluid of patients with endometriosis compared with controls and contribute to nociceptor sensitization and nerve growth [137,138,139,140,141]. IL-1β also induces endometrial stromal cells to produce NGF and BDNF, upregulates COX-2 expression, and increases PGE2 production [148]. PGE2 amplifies pain and inflammation while promoting local E2 synthesis, reinforcing a feed-forward loop that sustains lesion growth and symptoms [58,149,150]. Furthermore, IL-6 activates signaling pathways such as JAK/STAT3 that promote neuronal hyperexcitability and may support fibrosis and sustain local inflammation. Additional immune cells, including neutrophils and lymphocytes, further contribute to cytokine signaling and angiogenesis [151,152].

Overall, endometriosis-related pain exhibits both inflammatory and neuropathic features, reflecting this complex immune–neuronal interplay.

### 4.3. Central Sensitization and Pain Chronification

Pain severity in endometriosis does not correlate with lesion burden, reflecting variability in lesion innervation, peripheral sensitivity, and susceptibility to central sensitization [153,154]. Persistent pain after lesion removal further supports mechanisms beyond local disease [155].

Central sensitization arises from sustained nociceptive input, leading to increased excitability in spinal and brain pathways. It is common in endometriosis and is characterized by reduced pain thresholds and generalized hyperalgesia, with higher scores associated with more severe symptoms [156].

Neurobiological changes include altered brain connectivity, increased glutamate levels, and enhanced spinal signaling via upregulation of ion channels such as transient receptor potential vanilloid 1 (TRPV1), transient receptor potential ankyrin 1 (TRPA1), and voltage-gated sodium channel NaV1.7 [157,158,159]. These changes, along with synaptic remodeling and neuroinflammation, drive persistent pain states [160]. Overall, endometriosis-associated pain results from the interaction of peripheral inflammation, aberrant innervation, and central sensitization. Once established, this network can sustain chronic pain independently of lesion progression, highlighting the need for therapies targeting both peripheral and central mechanisms.

## 5. Hormone-Dependent Neuroinflammation in Endometriosis: Evidence for Bidirectional Interactions

Neuroinflammation is a key driver of pain in endometriosis, but its variability cannot be explained by lesion burden alone. Instead, it reflects bidirectional interactions between endocrine dysregulation and neuroimmune pathways that shape inflammation and pain persistence.

### 5.1. Hormonal Dysregulation as a Driver of Neuroinflammation

In endometriosis, hormonal imbalance is primarily local, characterized by estrogen dominance, P4 resistance, and potentially reduced androgen signaling. These shifts amplify immune–neuronal crosstalk, neurotrophin production, and nociceptor sensitization. Estrogen–prostaglandin signaling forms a self-reinforcing COX-2/PGE2 loop, although its activity varies by lesion type, with reduced expression in fibrotic deep lesions compared with ovarian lesions [109,161,162]. Inflammatory signals can further enhance local E2 production via NF-κB and TGF-β1, contributing to steroid receptor imbalance and reduced PR-B signaling [163].

Estrogen promotes neuroinflammation by enhancing macrophage–nerve interactions and neurotrophic signaling. Lesion-associated macrophages, enriched in ERβ, support nerve growth and sensitization, partly through mediators such as IGF-1 [164]. Estrogen also activates mast cells, increasing degranulation and histamine-mediated nociceptor sensitization, including TRPV1 activation [143,165].

Progesterone and androgens normally restrain inflammation, but this control is impaired in endometriosis. In healthy stromal cells, P4 suppresses NF-κB signaling and inflammatory mediators, whereas endometriotic cells fail to mount this response, consistent with P4 resistance [166,167]. Progestins such as dienogest can partially restore this anti-inflammatory effect and rebalance steroid receptor signaling, although responses vary by lesion type [168,169]. Inflammatory cytokines can further disrupt hormone responsiveness by reducing PR expression and altering AR signaling.

AR signaling has been proposed as an additional counter-regulatory influence on inflammatory activity in endometrial stromal biology, although direct evidence from endometriotic lesions remains more limited than for P4. Inflammatory cytokines can actively remodel the steroid receptor landscape in endometriosis-derived stromal cells, potentially compromising anti-inflammatory hormonal responses. In stromal cells from women with endometriosis, exposure to TNFα and IL-1β reduced P4 receptor expression and altered the expression of multiple nuclear receptors, including AR [170]. These findings indicate that inflammatory signaling itself can reshape the receptor milieu that determines responsiveness to both progestins and androgens.

### 5.2. Neuroinflammation as an Inducer or Amplifier of Hormonal Resistance

Accumulating evidence indicates that chronic inflammatory signaling within endometriotic lesions disrupts steroid hormone responsiveness. Inflammatory cytokines and immune-derived mediators converge on NF-κB-dependent transcriptional programs, epigenetic modifications, and alterations in steroidogenic pathways, promoting P4 resistance and relative estrogen dominance.

These processes establish a feed-forward interaction that sustains lesion activity and pain sensitization. Inflammation directly impairs PR expression and function. Pro-inflammatory cytokines, particularly TNF-α and IL-1β, suppress PR expression and can induce PR-B promoter hypermethylation, shifting signaling toward PR-A predominance and weakening P4 responses [170,171,172]. Additional epigenetic mechanisms, including microRNAs and histone modifications, likely reinforce this effect [90].

Disruption of P4/NF-κB crosstalk further sustains inflammation. In normal stromal cells, P4 inhibits NF-κB and downstream mediators such as IL-6 and COX-2, whereas in endometriotic cells this inhibitory control is lost, allowing persistent inflammatory signaling despite P4 exposure [173,174]. As a result, inflammation becomes self-sustaining and progressively reinforces functional P4 resistance. Inflammatory pathways also impair PR activity by altering co-regulators (e.g., FK506-binding protein 4, HIC-5/ARA55) and transcriptional partners such as FOXO1 and may favor expression of inhibitory isoforms such as PR-C [42,90,100]. In addition, pro-inflammatory cytokines may compete for shared transcriptional coregulators or disrupt the molecular bridges connecting PR to other transcription factors.

Collectively, these findings support a “vicious circle” model in which inflammation promotes P4 resistance, while impaired P4 signaling fails to restrain inflammatory transcriptional programs. NF-κB-driven cytokine production together with COX-2/PGE_2_-mediated steroidogenic changes appear central to this reciprocal amplification loop.

Inflammation also reshapes local steroidogenesis. Activated immune cells and platelets enhance E2 production via NF-κB and TGF-β1-dependent mechanisms, reinforcing estrogen dominance [163]. In parallel, as mentioned before, Erβ-biased signaling promotes COX-2/PGE2 activity and pro-survival transcriptional programs, supporting lesion persistence and inflammation [70,175,176].

Together, these findings support a reciprocal model in which inflammation drives P4 resistance and estrogen-dominant signaling, while impaired P4 activity fails to restrain NF-κB-mediated pathways. This self-sustaining loop maintains inflammatory activity, enhances neuroinflammation, and contributes to chronic pain in endometriosis.

## 6. Molecular Convergent Nodes at the Hormonal–Neuroinflammatory Interface in Endometriosis

Several molecular nodes consistently emerge from human-based evidence in endometriosis, supported by findings in patient-derived tissues, primary cell models, and clinical correlations with pain and fibrosis (Table 1). A common biological pattern is evident across lesion types: local estrogen dominance with P4 resistance, alongside sustained immune–neuronal interactions that drive peripheral sensitization and, in a subset of patients, chronic pain. We define the hormonal–neuroinflammatory interface by three criteria: regulation by E2 and/or P4 in human tissues, involvement in inflammation, innervation, or fibrosis linked to symptoms, and tractability for pharmacological targeting (Table 1) [177,178]. The candidate targets discussed below should be regarded as mechanistic hypotheses requiring validation in patient-derived multicellular systems incorporating epithelial, stromal, immune, and neural compartments, rather than as clinically validated nominations. Most current evidence derives from monocellular in vitro models, primarily isolated stromal or epithelial cells, which do not reproduce the spatial complexity of endometriotic lesions. We therefore frame each node not only by its mechanistic plausibility but also by the developmental stage at which clinical translation has stalled, the principal pitfalls, and the experimental work required to advance to the clinic.

This synthesis differs from existing reviews in three respects. First, evidence is stratified explicitly by lesion subtype (ovarian endometrioma, peritoneal lesion, DIE) and cellular compartment (stromal, epithelial, immune, neural), recognizing that the same molecular axis may be dominant in one subtype and silent in another [179,180]. Second, therapeutic maturity is annotated using a controlled vocabulary (Discovery → Preclinical → Phase I/II → Phase II terminated → Approved-other-indication → Off-label endometriosis) so that the distinction between mechanistically compelling and clinically actionable targets is immediately legible. Third, special attention is given to therapeutic options for adolescents and patients seeking fertility preservation, populations underserved by conventional GnRH-agonist-based protocols.

**Table 1:** Convergent druggable nodes at the hormonal–neuroinflammatory interface in endometriosis. Evidence is graded by lesion subtype, cellular compartment, and therapeutic maturity. Therapeutic-maturity vocabulary: Discovery (cellular evidence only); Preclinical (in vivo animal data); Phase I/II/III (active clinical trials); Phase II terminated (failed primary endpoint); Approved (other indication); Off-label endometriosis.

Target	Dominant Hormonal Input	Neuroinflammatory/Neurosensitizing Output	Human Evidence (Selected Effect Sizes; Lesion Specificity)	Therapeutic Maturity	Pros	Cons
PGE_2_–EP2/EP4 ↔ aromatase (CYP19A1) ↔ estradiol	Estradiol-dominant milieu reinforced by PGE_2_ signaling; inflammatory cytokines act upstream of PGE_2_	Sustained inflammatory mediator production; permissive microenvironment for innervation and sensitization	Human stromal cells: PGE_2_ induces aromatase activity ~19–44× in endometriosis-derived stromal cells [177]. Primary human ESC: EP2/EP4 antagonists reduce IL-1β-induced IL-6/IL-8 and suppress aromatase expression; macrophage EP2 expression higher in patients [177]. Letrozole + norethindrone reduces pelvic-pain VAS in adolescent and adult cohorts refractory to first-line progestins [181,182]. Lesion specificity: ovarian, peritoneal; reduced expression in fibrotic deep lesions [177,180].	AIs: Off-label (Phase II evidence). EP2/EP4 antagonists: Preclinical.	AIs address local intracrine estrogen production directly and are the most relevant option for adolescents and fertility-preserving patients in whom GnRH agonists are contraindicated. EP2/EP4 antagonism preserves systemic estrogen and may avoid menopausal sequelae [183].	AIs cause vasomotor symptoms, accelerated bone loss, and dyslipidemia; require add-back progestin [184]. Efficacy diminishes in fibrotic deep lesions where EP2/EP4 expression declines [180].
ERβ → NF-κB → CCL2	ERβ overexpression (up to 100-fold ectopic vs. eutopic); estradiol signaling bias	Macrophage recruitment; inflammatory amplification; stromal proliferation through macrophage–stromal feedback	Human cohort + cells (22 cases/14 controls): ERβ high → CCL2 via NF-κB; macrophages recruited; co-culture promotes ESC proliferation/clonogenicity [164] ERβ hypomethylation drives 34-fold stromal upregulation [179]. Lesion specificity: ovarian endometriomas predominant; also peritoneal [179].	Preclinical (PHTPP, ERB-041); SERMs (raloxifene): Phase II terminated for early pain recurrence [185,186].	Relative specificity for ectopic lesions; integrates hormonal and inflammatory signaling; nanoparticle co-delivery (PHTPP + disulfiram) shows lesion-selective effect in murine models without affecting normal endometrium [185].	Context-dependent signaling: agonists and antagonists can produce similar effects in different systems [70]. CCL2 redundancy in chemokine networks. Clinical translation of SERMs has so far failed [186].
IL-6/sIL-6R → STAT3 ↔ NF-κB (non-resolving inflammation → fibrosis)	Hormone–immune coupling via estradiol effects on cytokine environment in human ESCs)	Pro-fibrotic phenotype (collagen I, αSMA stress fibers), migration, persistent NF-κB activation	Human tissue + primary stromal cells (60 DIE cases vs. 32 controls): IL-6 transsignaling induces profibrotic phenotype in patient-derived ESCs but not in controls; impaired SOCS supports persistence; STAT3 inhibition reverses phenotype [178] DUSP2 downregulation in hypoxia amplifies IL-6/STAT3 [187]. Lesion specificity: DIE	Tocilizumab (anti-IL-6R): Approved (other indications); Endometriosis: preclinical [188,189]. STAT3 inhibitors: Discovery/Preclinical.	Disease-selective effect: trans-signaling preferentially active in endometriotic stromal cells. Anti-IL-6R agents already approved for rheumatologic indications, allowing repurposing trials.	STAT3 is essential for decidualization/implantation—systemic inhibition incompatible with fertility preservation. Pathway redundancy and feedback loops require combination strategies [190].
TRPV1/TRPA1 (neuronal + non-neuronal)	Inflammatory mediators (PGE_2_, TNF-α) shaped by estrogenic loops. E2 induces TRPV1 in human sensory neurons [191].	Nociceptor sensitization; cytokine and NO release from lesion cells; correlation with dysmenorrhea, dyspareunia, dyschezia	Human lesions + ESCs: TRPV1+ nerve-fiber density higher in ovarian endometrioma implants, correlates with dysmenorrhea VAS; PGE_2_ and TNF-α upregulate TRPV1 in EESCs; TRPV1 activation induces NO and IL-1β release [192]. In rectosigmoid DIE, TRPA1/TRPV1 upregulated and correlates with dysmenorrhea, dyspareunia, dyschezia [158]. Lesion specificity: ovarian, rectosigmoid DIE.	Phase II terminated (AMG517, ABT-102, LY3526318, GDC-0334) for off-target effects in non-endometriosis indications [193].	Strong mechanistic and clinical-correlational rationale for chronic pelvic pain. Peripherally restricted antagonists or topical/intralesional delivery would bypass the main systemic toxicity.	On-target hyperthermia and impaired heat perception (TRPV1 antagonists); insufficient efficacy or PK liabilities (TRPA1 antagonists). No endometriosis-specific trial to date [193].
NGF–TrkA/p75 axis	Estrogen-responsive tissue context; hormonal therapy reduces NGF in DIE [194].	Neurogenesis/nerve sprouting; peripheral sensitization; correlation with deep dyspareunia	Human pathology: DIE lesions show strong NGF, TrkA, p75 expression with high nerve density [195,196]. Stromal NGF correlates with dyspareunia severity and local nerve-bundle density. NGF stimulation in patient-derived ESCs increases COX-2 and PGE2 via Trk-dependent signaling [133]. Hormonal treatment reduces nerve fiber density and NGF/p75 expression in eutopic tissues and in DIE lesions [196]. Lesion specificity: DIE > peritoneal; bowel, rectovaginal.	Phase II terminated (tanezumab, anti-NGF)—unsuccessful in endometriosis pain (NCT00784693).	Mechanistically robust as a driver of neurogenic pain in DIE specifically. Strong dose–response correlation between NGF, nerve-fiber density, and dyspareunia (NCT00784693).	Tanezumab failed primary endpoint despite acceptable safety. Joint-related adverse events from anti-NGF program in osteoarthritis raise broader concerns [197]. Endometriosis pain is multimodal: single-pathway blockade unlikely to suffice.
BDNF–TrkB	Estradiol and IL-1β induce BDNF in human ESCs via ERK1/2	Central/peripheral pain maintenance signaling; correlation with dysmenorrhea VAS	Human cohort: serum and peritoneal-fluid BDNF higher in pain than in no-pain subgroups; ectopic lesion BDNF mRNA higher than eutopic/control; ESC BDNF inducible by estradiol or IL-1β and blocked by ERK inhibitor [130,198] BDNF/TrkB upregulated in ovarian endometrioma, with stage-dependent expression. Lesion specificity: ovarian; eutopic-tissue BDNF/TrkB correlates with dysmenorrhea	Discovery (no clinical agent in endometriosis).	Convergent estrogenic and inflammatory regulation positions BDNF as an integrative node. Eutopic-tissue expression suggests a non-invasive biomarker rationale.	Pan-Trk inhibitors (oncology) cause CNS adverse effects (cognitive impairment, mood disturbance, sleep disorders) reflecting BDNF/TrkB roles in synaptic plasticity [199]. Anti-BDNF antibodies failed to reduce endometriosis pain in murine models [134].
TNF-α → NK1R (TACR1) ↔ substance P signaling	Inflammatory TNF-α exposure; cytokine-driven receptor induction.	Neurogenic inflammation; lesion maintenance via neuropeptide signaling	Human matched samples + primary cells: TACR1/2 higher in ectopic vs. eutopic tissue; TACR1/NK1R correlates with peritoneal fluid TNF-α; TNF-α induces NK1R in stromal cells; substance P increases stromal cell viability, reversed by NK1R antagonist [200]	Approved (other indications): aprepitant for chemotherapy-induced nausea. Endometriosis: Discovery.	Aprepitant has an established safety profile, enabling rapid repurposing trials. Mechanistic link to lesion persistence beyond pain modulation.	Broad NK1R distribution in CNS and peripheral nervous system raises off-target concerns. No validated biomarker to identify neurogenic-inflammation-dominant patients. Tachykinin-receptor redundancy.
Mast cell activation (tryptase+) ↔ SCF (KITLG) in estrogenic contexts	Estrogen and progesterone modulate lesion–mast cell cross-talk; SCF in lesions promotes mast cell recruitment	Local cytokines (e.g., IL-6, IL-8); neuroimmune amplification near nerve fibers [7,135,136,165].	Human tissue (paired): endometriotic lesions show significantly higher tryptase+ mast cell density and SCF than eutopic [165]. Mast-cell conditioned media under hormonal conditions increases pro-inflammatory chemokines/cytokines in endometriotic epithelial/stromal cells [165]. Mast-cell density correlates with pain severity [7,135,136,165]. Lesion specificity: ovarian endometrioma (estrogen-driven mast-cell activation), peritoneal.	Cromolyn/ketotifen: Approved (other indications); endometriosis: Discovery.	Non-hormonal alternative preserving HPO-axis function—relevant for fertility-seeking patients. Disrupts the feed-forward E_2_–inflammation loop without systemic estrogen depletion [135].	Systemic MC stabilization may compromise innate immunity and wound healing [201]. Fibrotic shields in advanced lesions limit drug penetration [202,203] Functional redundancy with macrophages—likely insufficient as monotherapy

Legend: α-SMA—alpha smooth muscle actin; BDNF—brain-derived neurotrophic factor; cAMP—cyclic adenosine monophosphate; CCL2—C-C motif chemokine ligand 2; CYP19A1—aromatase; DIE—deep infiltrating endometriosis; EESC—ectopic endometrial stromal cells; EP2—prostaglandin E receptor 2; EP4—prostaglandin E receptor 4; ERβ—estrogen receptor beta; ERK—extracellular signal-regulated kinase; ESC—endometrial stromal cells; IL-1β—interleukin 1 beta; IL-6—interleukin 6; IL-8—interleukin 8; KITLG—KIT ligand; mRNA—messenger ribonucleic acid; NF-κB—nuclear factor kappa B; NGF—nerve growth factor; NK1R—neurokinin 1 receptor; NO—nitric oxide; p75—p75 neurotrophin receptor; PF—peritoneal fluid; PGE_2_—prostaglandin E_2_; SCF—stem cell factor; sIL-6R—soluble interleukin 6 receptor; SOCS—suppressor of cytokine signaling; STAT3—signal transducer and activator of transcription 3; TACR1—tachykinin receptor 1; TNF-α—tumor necrosis factor alpha; TrkA—tropomyosin receptor kinase A; TrkB—tropomyosin receptor kinase B; TRPA1—transient receptor potential ankyrin 1; TRPV1—transient receptor potential vanilloid 1.

### 6.1. The PGE_2_–EP2/EP4–Aromatase Axis

The PGE_2_–EP2/EP4–aromatase axis emerges as the most extensively documented convergent node in endometriosis. In patient-derived stromal cells, PGE_2_ induces aromatase activity 19- to 44-fold, establishing a self-reinforcing loop in which intralesional E_2_ stimulates COX-2 and PGE_2_ production, which in turn upregulates aromatase, generating further estrogen [176,177]. Selective EP2/EP4 antagonists abolish PGE_2_-induced cAMP elevation and IL-1β-driven IL-6/IL-8 secretion in primary endometriotic stromal cells, and macrophage EP2 expression is higher in patients than in controls [177].

Lesion specificity is non-uniform. PGE_2_ signaling is most active in early peritoneal and ovarian lesions; expression diminishes in advanced fibrotic DIE, where aromatase activity uncouples from EP2/EP4 input [180]. This stratification has direct therapeutic consequences: aromatase inhibitors and EP2/EP4 antagonists are mechanistically most appealing in earlier-stage disease, whereas heavily fibrotic lesions may require complementary anti-fibrotic strategies.

From a clinical-translation perspective, this axis carries particular relevance for two populations underserved by current first-line therapy. Adolescents with endometriosis refractory to combined oral contraceptives or progestins represent the first such group; GnRH agonists are typically contraindicated in this setting because of skeletal immaturity and the cumulative risk of bone mineral density loss during ongoing growth [204]. Adult women wishing to preserve fertility represent the second group; for these patients, prolonged ovarian estrogen suppression is undesirable. Letrozole 2.5 mg combined with low-dose norethisterone acetate has shown clinically meaningful reductions in pelvic pain VAS in both adolescent pilot studies and adult cohorts refractory to first-line progestins, with stable bone mineral density when add-back progestin is co-administered [181,182,205].

The EP2/EP4 antagonism strategy proposed by Arosh and colleagues, which suppresses local intralesional estrogen production without systemic estrogen depletion, offers a particularly attractive alternative for these populations and has demonstrated lesion regression without systemic menopausal effects in non-human primate models [183].

Despite this rationale, aromatase inhibitors (AIs) remain second-line because of their adverse effect profile: vasomotor symptoms, genitourinary atrophy, mood disturbances, accelerated bone resorption necessitating “add-back” therapy, and dyslipidemia compromising long-term cardiovascular health [184].

EP2/EP4 antagonists circumvent these limitations in principle, but their development is constrained by the broad physiological roles of PGE2 in renal hemodynamics, gastric mucosal protection, and bone remodeling, and by reduced EP2/EP4 expression in fibrotic lesions [180,183]. Endometriotic lesions undergo a “fibrotic shift” as they age, increasingly resembling scar tissue. In these advanced stages, the expression of EP2 and EP4 receptors appears to diminish, suggesting that while PGE2 antagonists may be highly effective for early-stage inflammatory disease, they may offer limited value for deep, chronic lesions [180].

Ultimately, this therapeutic target remains in a premature stage of development, currently navigating the complex transition from successful laboratory models to the rigorous clinical trials required to establish definitive human safety. Tissue-restricted delivery (e.g., intraperitoneal lavage formulations or lesion-targeted nanocarriers) and stratification of patients by lesion subtype and fibrotic content represent the principal experimental priorities for advancing this axis to the clinic [206].

### 6.2. The ERβ–NF-κB–CCL2 Axis

A substantial body of human evidence supports a central role for ERβ in endometriosis pathophysiology. Comparative immunohistochemical studies demonstrate a shift from ERα predominance in normal endometrium to marked ERβ overexpression and reciprocal downregulation of ERα [70,179]. Quantitative analyses in patient-derived stromal cells confirm up to 34-fold ERβ upregulation, driven in part by hypomethylation of the *ESR2* promoter [179]. Mechanistically, ERβ overexpression induces CCL2 production via NF-κB activation in endometriotic stromal cells, recruiting macrophages and establishing a pathogenic feedback loop in which macrophage–stromal interactions enhance proliferation and clonogenic potential [177]. ERβ further inhibits TNF-α-induced apoptosis and activates pro-survival programs, including SGK1 and Ras-like estrogen-regulated growth inhibitor signaling [70].

Lesion specificity matters. ERβ overexpression is most prominent in ovarian endometriomas, where epigenetic hypomethylation drives receptor imbalance, and substantial in peritoneal lesions; deep infiltrating lesions show more variable ERβ expression intermixed with fibrotic changes [179]. Within the cellular compartment, ERβ effects are stromal-dominant, although both nuclear and cytoplasmic localization are observed, suggesting genomic and non-genomic signaling components [70].

Clinical translation has been more difficult than the mechanistic case predicts. ERβ-selective agonists (ERB-041) and antagonists (PHTPP) both reduce lesion size in murine models, an apparent paradox that likely reflects context-dependent receptor signaling and differences in eutopic versus ectopic tissue expression [70]. Selective estrogen receptor modulators such as raloxifene showed efficacy in animal models but failed in patients: a randomized trial demonstrated earlier pain recurrence after surgical clearance, leading to early termination [186]. ERβ engages in extensive crosstalk with MAPK/ERK and PI3K/AKT pathways and with multiple inflammatory mediators, which may reduce therapeutic specificity and broaden off-target effects.

Recent advances in lesion-targeted drug delivery offer a route around these limitations. A dual-targeted liposomal nanosystem co-delivering the ERβ antagonist PHTPP and the pyroptosis inhibitor disulfiram has been reported to selectively accumulate in endometriotic lesions, suppress NF-κB-dependent inflammatory signaling, and reduce lesion size in murine models without affecting normal endometrium or ovarian function [185]. This approach combines ERβ inhibition with modulation of inflammasome-driven pathways, illustrating how spatial restriction may convert a context-dependent target into a clinically tractable one. Validation in patient-derived organoid–macrophage co-culture systems and in non-human primate models remains the principal next step.

### 6.3. The IL-6/sIL-6R–STAT3–NF-κB Axis

IL-6 trans-signaling through soluble IL-6 receptor (sIL-6R) and downstream STAT3 activation integrates inflammatory, fibrotic, and angiogenic processes in endometriosis. In a comparative study of 60 patients with DIE versus 32 controls, IL-6/sIL-6R induced a pro-fibrotic phenotype (increased collagen I deposition, αSMA stress-fiber formation, and enhanced migration) selectively in endometriotic stromal cells and not in stromal cells from healthy endometrium [178]. IL-6/sIL-6R signaling also activates NF-κB, while impaired SOCS3-mediated negative regulation supports sustained pathway activity. STAT3 inhibition or knockdown reverses the pro-fibrotic phenotype of the endometrium [178], establishing a direct mechanistic link between cytokine signaling and the fibrotic transformation that defines deep infiltrating disease.

Upstream amplification has also been characterized. Hypoxia-mediated downregulation of dual-specificity phosphatase-2 increases IL-6 expression in primary human stromal cells, leading to enhanced STAT3 activation and promoting endometriotic-cell proliferation and survival [187]. Lesion specificity favors DIE, consistent with the fibrotic and hypoxic microenvironment characteristic of this subtype.

Clinically, the IL-6 axis is unusually advanced relative to most endometriosis targets because anti-IL-6R agents (tocilizumab, sarilumab) are already approved for rheumatologic indications. Repurposing trials in endometriosis are mechanistically supported but complicated by the disease-specific selectivity of trans-signaling: the preferential activation of IL-6 trans-signaling in endometriotic stromal cells suggests that selective sgp130-Fc-based blockade, which spares classical IL-6 signaling, may offer a more favorable therapeutic window than broad receptor blockade. However, in a murine model of endometriosis, IL-6 blockade reduced STAT3 activation without decreasing lesion size and was associated with increased lesion attachment, suggesting context-dependent roles in invasion and adhesion. STAT3 itself is essential for physiological decidualization and embryo implantation, so systemic STAT3 inhibition is incompatible with fertility preservation [190]. Selective trans-signaling blockade and combinatorial strategies (e.g., with hormonal modulators) represent the most promising experimental directions.

### 6.4. TRPV1 and TRPA1 Channels

Human evidence implicates TRPV1 and TRPA1 in endometriosis-associated pain across multiple lesion types. In ovarian endometriomas, ectopic implants show a higher density of TRPV1-immunoreactive nerve fibers than control endometrium, and this density correlates with dysmenorrhea intensity [192]. Functional TRPV1 expression is documented in patient-derived ectopic endometrial stromal cells, where PGE_2_ and TNF-α upregulate channel expression, and TRPV1 activation drives nitric oxide and IL-1β release [192]. Women with endometriosis-related chronic pelvic pain show increased peritoneal expression of TRPV1, TRPA1, and SCN11A relative to pain-free controls without endometriosis. In rectosigmoid DIE, TRPA1 and TRPV1 are upregulated at both mRNA and protein levels in advanced-stage disease, with expression correlating with dysmenorrhea, dyspareunia, and dyschezia [148]. TRPV1/TRPA1 co-expression has been observed in association with macrophage polarization (CD86 and CD206) in pain-positive patients, linking channel activity to immune remodeling [207]. E_2_ itself induces TRPV1 expression in human sensory neurons, embedding TRP-channel sensitization within the broader estrogenic milieu of the lesion [191].

Clinical translation of TRP-channel modulators has been constrained by safety, efficacy, and drug-development barriers. Systemic TRPV1 antagonists (AMG517, ABT-102) have been limited mainly by on-target hyperthermia and impaired noxious-heat perception, reflecting the physiological role of TRPV1 in thermoregulation. TRPA1 antagonists (LY3526318, HX-100, GDC-0334) have faced more heterogeneous obstacles, including insufficient efficacy in proof-of-concept studies, potential toxicity, and complex pharmacokinetics. None of these candidates has been advanced into endometriosis-specific trials [191].

Future development would require, first, peripherally restricted antagonists or topical/intralesional delivery formulations that avoid central nervous system and thermoregulatory effects. Second, biomarker-guided patient stratification should be performed, for example, via peritoneal-fluid TRPV1/TRPA1 mRNA or by quantitative immunohistochemistry of nerve-fiber density, to identify patients in whom TRP-channel signaling is the dominant pain driver. Third, therapy could comprise the combination with anti-estrogenic or anti-inflammatory agents that interrupt the upstream estrogenic and cytokine inputs converging on TRP channel sensitization.

### 6.5. The NGF–TrkA/p75 Axis

The NGF–TrkA/p75 axis contributes to the neurogenic component of endometriosis-associated pain through lesion innervation and peripheral sensitization, although its expression is heterogeneous across disease phenotypes. DIE, particularly bowel and rectovaginal lesions, shows substantially higher nerve-fiber density than superficial peritoneal lesions. NGF, TrkA, and p75NTR have been detected in glandular and stromal compartments, but expression patterns are not uniform: compared with peritoneal lesions, deep infiltrating lesions exhibit higher stromal p75 expression, unchanged TrkA staining, and lower NGF-positive staining in glandular and stromal compartments [195,196]. In posterior-compartment endometriosis involving the cul-de-sac or uterosacral ligaments, higher NGF and TrkA expression has been reported in women with deep dyspareunia, with stromal NGF correlating with both dyspareunia severity and local nerve-bundle density [120]. In patient-derived endometriotic stromal cells, NGF stimulation enhances COX-2 expression and PGE_2_ secretion through a Trk-dependent mechanism, providing a direct molecular link between neurotrophin signaling and the prostaglandin axis. In rectovaginal septum DIE, hormonal therapy is associated with reduced nerve-fiber density and NGF expression, whereas TrkA expression remains unchanged [194].

Clinical translation has nevertheless stalled. The most direct endometriosis-specific attempt was tanezumab, a humanized anti-NGF monoclonal antibody evaluated in a phase 2 randomized double-blind placebo-controlled trial. Tanezumab was administered as a single 15 mg intravenous dose; the primary endpoint was the change from baseline to Week 8 in average daily endometriosis pain. Recruitment was discontinued in December 2009 with all enrolled patients completing scheduled visits; the trial did not demonstrate a consistent analgesic advantage over placebo on endometriosis pain endpoints, and no further endometriosis-specific clinical development of NGF inhibitors has been pursued (NCT00784693). Two factors likely explain the limited efficacy. First, endometriosis pain is multimodal, combining lesion-driven inflammation, peripheral sensitization, neuropathic features, and central pain amplification. Therefore, single-pathway blockade is unlikely to suffice. Second, joint-related adverse events from the broader anti-NGF program in osteoarthritis raised safety concerns that have constrained the development of next-generation agents in non-oncological pain indications.

Productive next steps include patient stratification by lesion subtype (selecting DIE with documented hyperinnervation), combination therapy with hormonal or anti-inflammatory agents to address the multimodal pain phenotype, and development of selective TrkA antagonists with improved safety profiles. The strong correlation between NGF, nerve-fiber density, and dyspareunia in DIE [133,194] justifies re-evaluating this axis in targeted patient groups.

### 6.6. The BDNF–TrkB Axis

Beyond NGF, the BDNF–TrkB pathway is a key neurotrophin signaling axis increasingly linked to endometriosis-associated pain. Serum and peritoneal-fluid BDNF concentrations are higher in pain-associated than in pain-free patients [130]. At the tissue level, BDNF mRNA expression is increased in ectopic lesions relative to eutopic and control endometrium and correlates with pain intensity. In endometriotic stromal cells from patients, E_2_ and IL-1β stimulate BDNF mRNA and protein expression, and ERK1/2 inhibition attenuates this response, implicating convergent estrogenic and inflammatory inputs [130]. In ovarian endometrioma, BDNF and TrkB are more highly expressed in ectopic than in eutopic or normal endometrium, with greater expression in stage-IV disease compared with stages II–III, and eutopic-tissue BDNF/TrkB mRNA correlates with dysmenorrhea VAS scores [198]. Predominant ERβ, BDNF, and TrkB expression in ectopic versus paired eutopic ovarian tissue suggests functional crosstalk between ERβ-biased estrogen signaling and the BDNF–TrkB pathway [198].

Translation into endometriosis therapy remains limited. The available human evidence is observational, demonstrating elevated expression and pain associations in ovarian endometrioma but not establishing BDNF–TrkB as the dominant driver in all patients. The principal translational concern is biological: BDNF–TrkB signaling participates in synaptic plasticity, cognition, and mood regulation, and clinically used pan-Trk inhibitors (developed for oncology indications) are associated with central nervous system adverse effects, including dizziness, cognitive impairment, mood disorders, and sleep disturbance [199]. Preclinical endometriosis data are not uniformly supportive: selective anti-BDNF blockade failed to reduce endometriosis-associated pain in a murine model, in contrast with the reduction observed with anti-NGF strategies in the same study [134]. Therefore, BDNF–TrkB appears to contribute to endometriosis-associated pain in a phenotype-dependent and lesion-subtype-dependent manner, while its pharmacological targeting remains constrained by biological complexity. Eutopic-tissue BDNF/TrkB expression may have greater near-term value as a non-invasive biomarker of pain severity than as a direct therapeutic target.

### 6.7. TNF-α-Driven NK1R (TACR1) and Substance P Signaling

TNF-α-mediated upregulation of NK1R (TACR1) provides a mechanism through which inflammatory signaling facilitates neurogenic pathways in endometriosis. In matched human samples and primary stromal cell studies, TACR1 and TACR2 expression are significantly increased in ectopic compared with eutopic endometrial tissue, and NK1R expression correlates with peritoneal fluid TNF-α concentrations [200]. In vitro, TNF-α directly induces NK1R expression in endometrial stromal cells, establishing a permissive environment for substance P signaling. Functional assays demonstrate that substance P enhances stromal-cell viability, and this effect is reversed by NK1R antagonists, supporting a role for the pathway in lesion persistence beyond pain modulation [200]. Substance P/NK1R signaling is broadly recognized as a key mediator of neurogenic inflammation, and the peritoneal microenvironment in endometriosis is characterized by immune dysfunction and increased TNF-α-driven activity that promotes lesion survival.

NK1R is widely expressed in the central and peripheral nervous systems, where it contributes to physiological pain modulation and inflammatory signaling. This broad distribution limits therapeutic specificity and raises concerns about off-target neurological effects. In addition, neuropeptide signaling exhibits functional redundancy involving multiple tachykinins (substance P, neurokinin A, neurokinin B) and three receptor subtypes (NK1R, NK2R, NK3R), which may reduce the efficacy of selective NK1R blockade. The complex interplay of immune cells, nerve fibers, and stromal cells within the lesion microenvironment further complicates the prediction of therapeutic responses, consistent with the broader heterogeneity of immune receptor expression in endometriosis.

Repurposing represents the most actionable translational route. NK1R antagonists such as aprepitant and netupitant have established safety profiles in chemotherapy-induced nausea and vomiting, allowing rapid evaluation in carefully designed endometriosis trials. The principal experimental requirements are validated biomarkers to identify patients with predominant neurogenic inflammation (e.g., peritoneal-fluid TNF-α and tissue NK1R/Substance P quantification) and animal models that assess chronic neurogenic pain rather than acute nociception. Combinatorial approaches with anti–TNF-α agents are mechanistically attractive given the TNF-α-driven receptor induction documented in human samples [200].

### 6.8. Mast-Cell Activation and the SCF–KIT Axis

Mast cells are increased in endometriotic lesions across subtypes and are co-localized with sensory nerve fibers, providing a structural substrate for neuroimmune amplification. Paired-tissue analyses demonstrate significantly higher tryptase-positive mast-cell density and stem-cell factor (SCF/KITLG) expression in ectopic versus eutopic endometrium [7,165]. Mast-cell density correlates with pelvic-pain severity [7]. In vitro, mast-cell conditioned media obtained under hormonal-treatment conditions increase pro-inflammatory cytokine output (IL-6, IL-8, MCP-1) in endometriotic epithelial and stromal cells, while estrogen drives mast-cell activation in ovarian endometriomas, integrating mast-cell biology into the broader estrogenic microenvironment of the lesion [165].

From a translational perspective, mast-cell stabilization is attractive for a specific clinical scenario: patients seeking symptomatic relief while preserving HPO-axis function and reproductive potential [135]. Mast-cell stabilizers such as cromolyn sodium and ketotifen are approved for other indications and are mechanistically positioned to disrupt the feed-forward E_2_–inflammation loop without systemic estrogen depletion [135]. By inhibiting degranulation, this approach reduces local cytokine output (limiting downstream aromatase upregulation) and blocks the release of mast-cell-derived proteases (tryptase, chymase) and VEGF which contribute to tissue invasion and neovascularization.

Three pharmacological and biological limitations constrain clinical translation. First, systemic mast-cell inhibition may compromise innate immune surveillance and homeostatic tissue regeneration, given the role of mast cells in pathogen defense and wound healing [201]. Second, the progression of endometriosis toward dense fibrosis creates a physical barrier, “fibrotic shields”, that may prevent therapeutic agents from reaching mast-cell populations sequestered in deep, established lesions [203]. Third, functional redundancy with other resident leukocytes, particularly macrophages, may sustain the inflammatory cascade in the event of mast-cell inhibition, suggesting that monotherapy is unlikely to suffice. Combinatorial strategies pairing mast-cell stabilization with anti-fibrotic or macrophage-targeted approaches, together with topical or intralesional delivery to circumvent the fibrotic-shield problem, represent the most promising experimental directions.

## 7. Discussion

Human-based mechanistic studies of endometriosis converge on a single integrative model: disease persistence and pain are not parallel manifestations of distinct processes but coupled outputs of a self-reinforcing loop in which local estrogen dominance, progesterone resistance, and neuroinflammation reciprocally amplify each other. The eight molecular nodes summarized in Section 6—spanning prostaglandin–aromatase signaling, ERβ-driven NF-κB activation, IL-6/STAT3-mediated fibrosis, neurotrophin and TRP-channel sensitization, and mast-cell-dependent neuroimmune amplification—operate as a partially overlapping networks rather than as independent pathways [7,130,133,135,136,158,164,165,175,176,177,178,179,180,181,182,187,192,194,195,196,198,200]. This framing reconciles two long-standing clinical observations that an estrogen-only or lesion-burden model cannot explain the dissociation between lesion size and pain severity, and the persistence of symptoms after hormonal suppression or surgical clearance in a substantial subset of patients.

Heterogeneity in endometriosis is not a methodological nuisance but a primary biological feature with direct consequences for trial design [5,60,65,129]. Stratification by lesion subtype reveals distinct molecular signatures: ovarian endometriomas display the highest ERβ overexpression and estrogen-driven mast-cell activation; deep infiltrating lesions are characterized by IL-6/STAT3-driven fibrosis, hyperinnervation, and elevated NGF expression, often with disproportionate pain severity relative to lesion size [60]; peritoneal lesions show intermediate profiles, with prominent COX-2/PGE_2_ activity in earlier-stage disease that diminishes as lesions become fibrotic. Cellular compartmentalization adds a second axis: stromal cells dominate steroid-receptor signaling and progesterone resistance; epithelial cells contribute lesion-persistence programs; immune–neuronal interactions sustain pain amplification. A third axis, pain phenotype, overlays these substrates: inflammatory pain in lesion-active disease, neuropathic features in densely innervated lesions, and central sensitization that may persist after lesion clearance. The practical implication is that single-target trials enrolling unselected populations will likely continue to fail, as exemplified by the unsuccessful tanezumab phase 2 trial in endometriosis pain. Future trials should pre-specify lesion subtype, treatment history, and pain phenotype as inclusion criteria.

The eight targets discussed above occupy substantially different positions on the translational gradient and conflating them risks overstating clinical readiness. Two strategies are clinically actionable in the near term. AI are off-label but supported by Phase II data in adolescent and adult populations refractory to first-line progestins, and their established adverse-effect profile [183] is now well managed with progestin add-back. Anti-IL-6R agents are approved for rheumatologic indications and are mechanistically positioned for repurposing trials in DIE, where IL-6 trans-signaling is preferentially active in patient-derived stromal cells [170]. Three further strategies are at intermediate readiness: EP2/EP4 antagonists with strong preclinical data in non-human primates [182], lesion-targeted ERβ-modulating nanocarriers, and repurposed mast-cell stabilizers [185]. Four targets remain mechanistically compelling but speculative for endometriosis: TRPV1/TRPA1, NGF–TrkA, BDNF–TrkB, and NK1R/Substance P. For each, human evidence demonstrates lesion-level upregulation and clinical-correlational pain associations, but every dedicated drug-development program has been constrained by on-target (TRPV1 antagonists) or off-target (pan-Trk inhibitors) toxicity, failed primary endpoints (tanezumab), or absence of any endometriosis-specific clinical evidence (NK1R). For these targets, the appropriate framing is hypothesis-generating: peripherally restricted compounds, biomarker-guided stratification, and combinatorial designs are needed before further clinical investment is warranted.

This review has several limitations. First, most molecular evidence derives from monocellular in vitro systems—primarily isolated stromal or epithelial cells—that cannot reproduce the multicellular architecture of endometriotic lesions; emerging co-culture, organoid, and microfluidic platforms partially address this gap but rarely incorporate functional innervation, leaving the neural component of the proposed feedback loops experimentally underspecified [208,209,210,211,212,213].

Second, reliance on surgically obtained samples introduces a substantial selection bias: surgical cohorts are enriched for advanced or treatment-resistant disease, which probably overestimates the magnitude of molecular alterations and limits applicability to earlier-stage disease, where therapeutic intervention may be most effective. Third, the absence of longitudinal sampling prevents reconstruction of disease evolution and the temporal dynamics of the pathways involved; virtually all human studies in this field are single-time-point and therefore unable to distinguish driver mechanisms from compensatory or downstream changes. Fourth, prior or ongoing hormonal therapy modulates gene expression, inflammatory signaling, and DNA methylation profiles in endometriotic tissue, complicating the separation of disease-related from treatment-related changes; few studies systematically document treatment exposure at sampling, and fewer still stratify analyses accordingly. Fifth, methodological heterogeneity in tissue sampling, immunohistochemical markers, and pain assessment instruments contributes to the variability in reported nerve fiber density and neurotrophins expression data, limiting cross-study integration. Although molecular profiles differ across epithelial and stromal compartments and between lesion subtypes, their relationship to clinical pain phenotypes remains insufficiently defined. Collectively, these constraints suggest that the targets identified here should be viewed as mechanistic hypotheses backed by human evidence, rather than clinically validated candidates.

Three research priorities follow from this synthesis. First, validation of the proposed targets requires patient-derived multicellular systems incorporating epithelial, stromal, immune, and—critically—neural compartments. Organoid–macrophage–mast-cell co-cultures, microfluidic platforms with microvascularization and partial innervation, and where, ethically feasible, non-human-primate models of endometriosis, offer increasingly tractable substrates. Second, longitudinal cohorts with documented treatment exposure are needed to distinguish driver from downstream mechanisms and to identify pathways whose activity predicts persistent pain after surgical or hormonal intervention. Such cohorts also enable the development of non-invasive biomarkers such as peritoneal-fluid TNF-α, circulating BDNF, or eutopic-tissue neurotrophin expression, that could stratify patients for mechanism-targeted trials. Third, trial designs should explicitly accommodate disease heterogeneity: enrichment by lesion subtype (e.g., NGF–TrkA blockade restricted to bowel/rectovaginal DIE with documented hyperinnervation; IL-6R blockade restricted to fibrotic deep disease), combinatorial regimens that address the multimodal pain phenotype, and adaptive designs allowing early identification of responder subgroups. The therapeutic implications extend beyond drug discovery: the convergent-node perspective offers a basis for revisiting the clinical management of patients with pain refractory to GnRH analogues, particularly adolescents and women seeking fertility preservation, in whom systemic estrogen suppression carries the greatest cumulative cost.

## 8. Conclusions

Breaking the current therapeutic deadlock in endometriosis requires moving beyond systemic hormonal suppression toward an interface-targeted approach. Our analysis highlights that P4 resistance and neuroinflammation are not parallel events but coupled components of a self-sustaining feedback loop, in which ectopic lesions acquire substantial endocrine and neural autonomy from systemic hormonal regulation.

The future of clinical management may lie less in global suppression of the estrogenic axis than in selective modulation of the lesional microenvironment. Identification of convergent druggable pathways may support more precise pharmacological strategies. However, clinical translation will require validation in patient-derived multicellular systems and in carefully stratified clinical trials. Therapeutic success will depend on the capacity to transition from standardized treatments to mechanism-targeted interventions that restore local homeostasis with the long-term goal of addressing the underlying drivers of endometriosis-associated pain rather than its symptoms alone.

## Data Availability

No new data were created or analyzed in this study. Data sharing is not applicable to this article.
